# Photodynamic Therapy (PDT) with Chemotherapy for Advanced Lung Cancer with Airway Stenosis

**DOI:** 10.3390/ijms161025466

**Published:** 2015-10-23

**Authors:** Masakazu Kimura, Kuniharu Miyajima, Masakazu Kojika, Takafumi Kono, Harubumi Kato

**Affiliations:** Department of Thoracic Surgery, Niizashiki Central General Hospital, 1-7-2 Touhoku, Niiza, Saitama 352-0001, Japan; E-Mails: miyajima@wb3.so-net.ne.jp (K.M.); mahalo.1@rd5.so-net.ne.jp (M.K.); t-kono1@kd5.so-net.ne.jp (T.K.); kaharu@gol.com (H.K.)

**Keywords:** advanced lung cancer, photodynamic therapy, chemotherapy, bronchial obstruction, bronchial stenosis

## Abstract

Intractable advanced lung cancer can be treated palliatively with photodynamic therapy (PDT) combined with chemotherapy to remove central and peripheral (lobar or segmental bronchi) bronchial stenosis and obstruction. We present data for 12 (eight men, four women) consecutive patients with 13 advanced non-small cell lung carcinomas in whom curative operations were contraindicated, who underwent PDT combined with chemotherapy for local control of the intraluminal lesions. The mean age was 73.3 years (range, 58–80 years), and the stages of cancer were IIA–IV. The median stenosis rates before treatment, one week post-treatment, and one month post-treatment were 60% (range, 30%–100%), 15% (range, 15%–99%), and 15% (range 15%–60%), respectively. The mean and median survival times were 9.3 and 5.9 months, respectively. The overall 1-year survival rate was 30.0%. No PDT-related morbidity or mortality occurred. In this single-institution study, all patients experienced improved symptoms and quality of life at one week after treatment; furthermore, an objective response was evidenced by the substantial increase in the openings of the bronchial lumen and prevention of obstructive pneumonia. Therefore, PDT with chemotherapy was useful and safe for the treatment of bronchial obstruction.

## 1. Introduction

The Ministry of Health, Labor and Welfare in Japan estimated that there were approximately 112,000 new cases of and 70,300 deaths from lung cancer in 2011.

Approximately 30% of patients with lung cancer also have airway stenosis [1]. Although the standard treatment for airway stenosis is surgical resection and reconstruction, patients with central airway stenosis due to lung cancer are often poor surgical candidates based on either physiological or oncological criteria [1,2]. Therefore, these patients often require palliative intervention particularly for obstruction or bleeding that may result in death. Treatment should support for obstructions such as endoluminal, extraluminal, or infiltrating obstruction. Endobronchial treatment is commonly associated with quick symptom improvement and limited side effects. Endoluminal obstructions influence the treatment effect of external beam radiation therapy, photodynamic therapy (PDT), laser therapy, or brachytherapy [3].

PDT is recommended as curative for only small lesions (≤1.0 cm in diameter) in the 2003 American College of Chest Physicians guidelines [4]. However, Usuda *et al*. reported based on their experience with 264 lesions, that PDT treatment with Laserphyrin^®^ prevents recurrence after complete remission (CR), regardless of tumor size [5] (maximum diameter in the longitudinal axis: <0.5 cm, 56 lesions; 0.5–0.9 cm, 124 lesions; 1.0–2.0 cm, 50 lesions; and >2.0 cm, 34 lesions). Although the CR rates were 94.6%, 93.5%, 80%, and 44.1%, respectively, the CR + PR rates were 100% for all lesion sizes [6].

PDT or neodymium-doped yttrium aluminum garnet (Nd:YAG) laser treatment was also effective in terms of tumor response in 73% and 76% of patients, respectively, with tumors in the lobar or segmental bronchi [7]. Furthermore, PDT appears to be useful for treatment of obstructions in the lobar and segmental bronchi, when compared with Nd:YAG laser or radiotherapy, which usually affects all of the parts of the wall and damages collagen fibrils and smooth muscle. PDT is more selective than Nd:YAG laser and radiotherapy, sparing the collagen fibrils and enabling healing predominately by regeneration [8].

In this report, we evaluate the safety and efficacy of PDT with chemotherapy for advanced lung cancer with airway stenosis. Symptoms and quality of life (QOL) were improved, and there was an objective response to treatment, with increased openings of the bronchial lumen and prevention of obstructive pneumonia. Furthermore, the treatment appeared to be safe, when combined with standard therapeutic methods.

## 2. Results and Discussion

### 2.1. Patient and Tumor Characteristics

The mean age of the 12 patients (eight men, four women) was 73.3 years (range 58–80 years), and the lung cancer stages were stage IIA in one patient, IIB in one patient, IIIA in two patients, IIIB in three patients, and IV in five patients. The histopathological diagnoses of the 13 lesions in the 12 patients were squamous cell carcinoma in seven patients, adenocarcinoma in three patients, large cell neuroendocrine carcinoma in one patient, and giant cell carcinoma in one patient. The primary locations of the tumors were the right upper lobe bronchus in four patients, truncus intermedius in three patients, right main bronchus in two patients, carina in one patient, right basal bronchus in one patient, and left upper lobe bronchus in one patient.

### 2.2. Treatment Results

**Figure 1:**
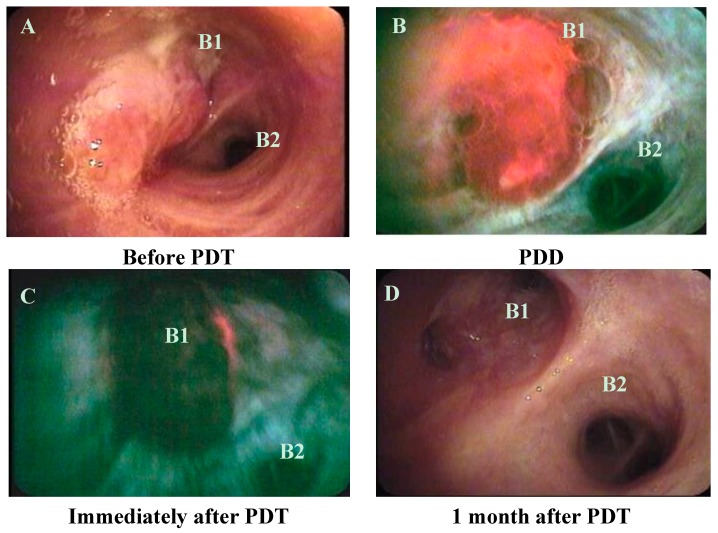
Bronchoscopic appearance of case 1 (**A**) before photodynamic therapy (PDT), when squamous cell carcinoma can be seen in the left upper lobe bronchus (left B1 and B2); (**B**) during photodynamic diagnosis (PDD) with the SAFE-3000 before PDT; (**C**) immediately after PDT; and (**D**) 1 month after PDT (left B1 and B2 are visible).

**Figure 2:**
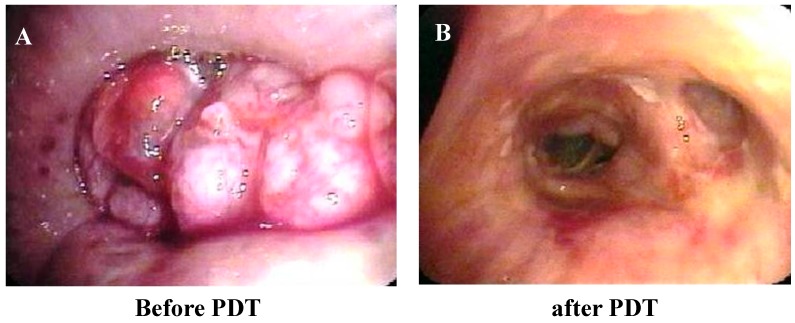

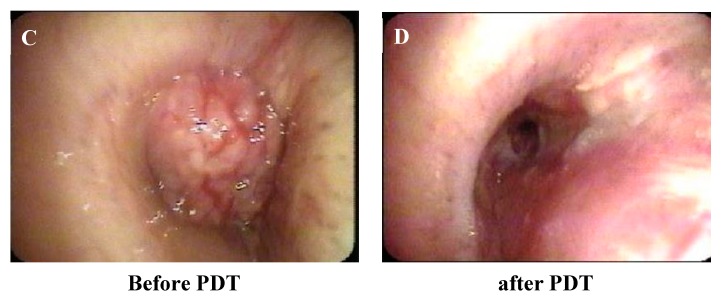
Bronchoscopic appearance (**A**) before photodynamic therapy (PDT) in case 2; (**B**) after PDT in case 2; (**C**) before PDT in case 12; (**D**) after PDT in case 12.

**Figure 3:**
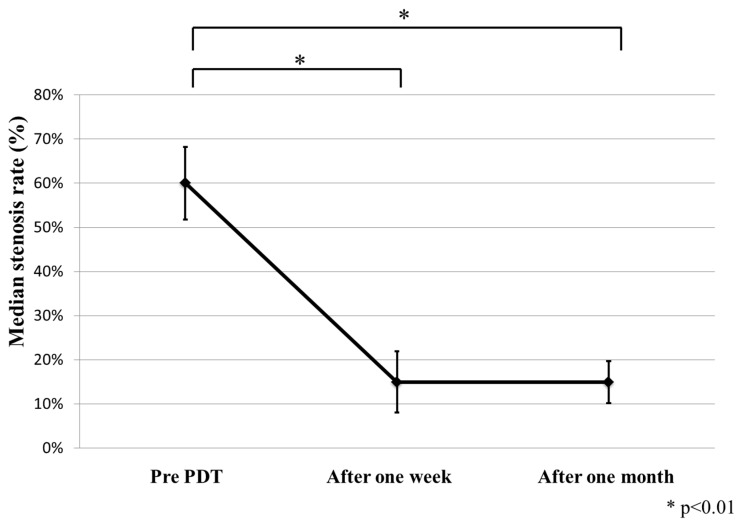
Significant improvement in the median airway stenosis rate 1 week (*p* = 0.0003) and 1 month (*p* = 0.0016) after photodynamic therapy (PDT), compared with before surgery.

The median stenosis rates before PDT, one week after PDT, and one month after PDT were 60% (range 30%–100%), 15% (range, 15%–99%), and 15% (range, 15%–60%), respectively (*p* = 0.0003, before PDT compared with one week after PDT; *p* = 0.0016, before PDT compared with one month after PDT; Figure 1, Figure 2 and Figure 3).

The median Karnofsky scores (KS) before and one month after PDT were 85 (range, 20–100) and 100 (range, 90–100), respectively (Table 1). The median vital capacity and forced expiratory volume in 1 s in the three patients who underwent pulmonary function tests were 1.93 and 1.28 L, respectively, before PDT; and 2.58 and 1.67 L, respectively, two months after PDT.

At one month after PDT, symptoms and QOL were improved in all of the patients, and there was an objective response to treatment, as indicated by a substantial increase in the openings of the bronchial lumen and the prevention of obstructive pneumonia. The following grades (CTCAE v4.02) of chemotherapy side effects were experienced: Grade 1 in eight patients, Grade 3 in three patients, and Grade 4 in one patient. There was no PDT-related morbidity or mortality.

**Table 1:** Backgrounds and an effect of PDT.

Case	Age	Patho	c-Stage	Chemotherapy	Location	Probe	before PDT S.R.	1W after PDT S.R.	1M after PDT S.R.	Survival (Day)	Outcome	CTCAE v4.0 (Grade)	KS (before)	KS (after)
1	77	Sq	IIA	CBDCA + TS-1	LU (B1 + 2)	F and R	45%	15%	15%	224	Death	1	90	100
2	74	LCNEC	IIIB	NDP + DOC	R Int	F and R	100%	60%	15%	160	Death	1	20	100
3	74	Sq	IIB	CBDCA + TS-1 CBDCA + VNR	R Int	F and R	60%	15%	30%	638	Death	3	90	90
4	69	Ad	IV	TS-1	RM	F and R	30%	15%	30%	61	Death	1	20	90
5	75	Sq	IV	CDDP + DOC	RM	F and R	30%	15%	45%	93	Death	3	80	90
6	74	Giant	IV	CDDP + DOC	RL	F and R	100%	99%	60%	425	Death	1	70	100
7	72	Sq	IV	CDDP + DOC	Carina	F	R 30% L 60%	R 15% L 15%	R 15% L 15%	196	Death	3	90	100
8	58	Sq	IIIB	CBDCA + TS-1	RU (B2,3)	F and R	45%	30%	15%	614	Alive	1	90	100
9	77	Sq	IIIA	NDP + DOC	RU	F	99%	15%	15%	38	Death	4	90	100
10	78	ad	IIIA	CBDCA + PEM + Bev CBDCA + PTX + Bev	RU(B3)	F and R	60%	30%	60%	159	Death	1	100	100
11	80	ad	IV	CBDCA + PEM + Bev	RU	F and R	99%	15%	30%	89	Alive	1	80	90
12	70	sq	IIIB	CBDCA + nab-PTX CBDCA + TS-1	R Int	F and R	100%	30%	15%	195	Alive	1	80	100

RM, Right main bronchus; RU, Right upper bronchus; R Int, Right bronchus intermedius; RL, Right lower bronchus; LU, Left upper bronchus; F, Frontal; R, radial; S.R, Stenosis rate; KS, Karnofsky score; Ad, adenocarcinoma; Sq, squamous cell carcinoma; Giant, Gaiant cell carcinoma.

### 2.3. Tumor Responses on Computed Tomography

**Figure 4:**
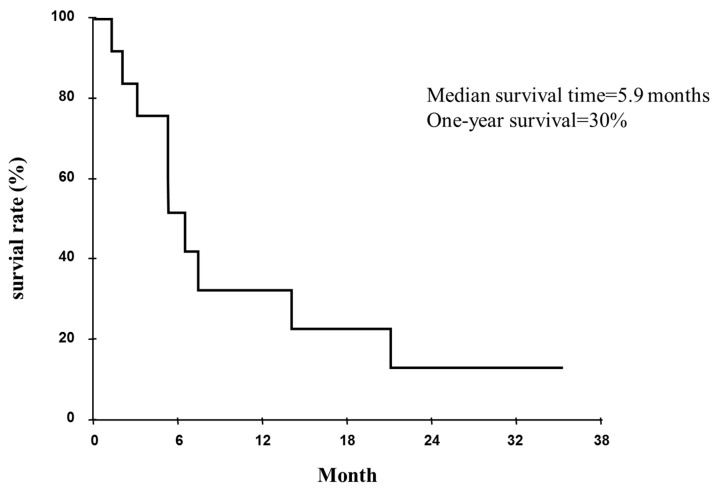
Survival curve for cases of advanced lung cancer with airway stenosis, as measured from the time of photodynamic therapy (PDT), the mean survival time (MST) was 177.5 days.

Six patients experienced a partial response, three patients had stable disease, and three patients had progressive disease. The mean survival time was 5.9 months (Figure 4). The overall one-year survival rate was 30.0%.

### 2.4. Discussion

Of the treatment options for central airway stenosis, we chose not to use YAG laser therapy, brachytherapy, or extra beam radiation therapy for a number of reasons. High-power laser vaporization (Nd:YAG) is used for local adjuvant therapy in combination with radiotherapy, chemotherapy, or airway stenting and primarily targets tumors occurring in large airways, from the trachea to the entries of segmental bronchi [9]; however, Nd:YAG laser therapy can cause complications such as hemorrhage, perforation, fistulae formation, a flame in the airway, and pneumothorax. Brachytherapy can cause complications such as hemoptysis (which may result in death), bronchial necrosis, airway fistulas to the neighboring structures, fibrotic stenosis, and radiation bronchitis, which are due to the actual radiation effects. Radiation pneumonitis is the primary complication of extra beam radiation-therapy, and interstitial lung disease (ILD) is regarded in the Japanese Society for Therapeutic Radiation and Oncology clinical guidelines as a contraindication for stereotactic body radiation therapy [10] because the most dangerous complications are massive bleeding from blood vessel perforations or ILD. In the present study, PDT was performed safely, without perforation or ILD. Although PDT and conventional Nd:YAG laser therapy are equally effective and safe for airway obstructions in advanced non-small cell lung cancer, PDT results in better survival and has a longer time to treatment failure [11].

In the present study, bloody sputum production and obstructive pneumonia improved within 1 week after PDT, and cough, sputum, and dyspnea production improved within one month after PDT. We believe that the speed of opening the airway with PDT clears the obstructive pneumonia. Because symptoms were improved with the reduction of the endobronchial tumor, KS also improved.

The combination of PDT and chemotherapy was justified to improve the airway obstruction symptoms and KS in these patients with lung cancer airway obstruction. PDT attempts to control internal bronchial tumor growth, and the additional systemic chemotherapy provides certain benefits for survival with advanced lung cancer. Similarly, Hong *et al*. reported that treatment with systemic chemotherapy and PDT might produce a synergistic effect that improves survival and QOL for patients with advanced bile duct cancer [12]. In an *in vivo* study using a nude mouse model, the tumor necrotic area, percentage of apoptosis-positive cells, and percentage of vascular endothelial growth factor (VEGF)-producing cells were significantly higher in the group treated with a therapy with combined chemotherapy and PDT than in the groups treated with chemotherapy alone or PDT alone [13].

It has been reported that PDT induces oxidative stress, localized inflammation, and vascular injury within treatment fields, and these responses resulted in an increase in the expression of angiogenesis factors and cytokines [14]. Ferrario *et al*. reported that VEGF inhibitors or cyclooxygenase-2 increased the effectiveness of PDT [15]. Tumor bleeding disappeared just after PDT in the present study. Because of the early stage, PDT-induced microvascular damage and the resulting hypoxia could activate molecular events that lead to the increased expression of VEGF within the treated tumor tissue.

The limitations of this study are the relatively small number of patients, retrospective nature, and single institution. Further prospective randomized trials are needed to compare the ability of this technique with other additional procedures to prevent symptoms of airway obstruction and improve QOL. If the results are supported by additional studies, we recommend that these procedures be adopted as standard therapy.

## 3. Experimental Section

### 3.1. Patients

Data were retrospectively collected for 13 lesions of the 12 patients with complicated (e.g., ILD) advanced non-small cell lung cancer or endoluminal malignant obstructive lesions who underwent PDT combined with chemotherapy at Niizashiki Central General Hospital between December 2010 and September 2014. The patients were diagnosed using bronchoscopy, and the diagnosis of non-small cell lung carcinoma was histopathologically confirmed. The majority of the patients had been investigated, diagnosed, and determined to be ineligible for surgical resection prior to referral to our team.

### 3.2. Photosensitizer and Laser Unit

NPe6 (talaporfin sodium, Laserphyrin^®^, Meiji Seika, Tokyo, Japan) is a second-generation, water-soluble photosensitizer with a molecular weight of 799.69 and a chlorine annulus. Its maximum absorption peak is at a wavelength of 407 nm, and there is a second peak at 664 nm [6,14,16,17]. Laserphyrin^®^ has a high tumor affinity and is excited by visible red light with a longer wavelength of 664 nm, enabling deeper and superior penetration into living tissues [6,14,16]. A diode laser (Matsushita Electric Industrial Co., Osaka, Japan) emitting continuous-wave laser light at a wavelength of 664 nm is used as the light source for excitation of Laserphyrin^®^ [6,14,16,17]. Two types of fiber-optic tips can be used: a straight or cylindrical/radial type. Although we usually use the straight-type fiber-optic tip, we use the radial type for tumors that have spread to the bronchial wall, inserting this type of tip into the tumor.

### 3.3. Treatment Protocol

Laserphyrin^®^ (40 mg/m^2^) was intravenously administered. Four hours after administration, PDT was performed using bronchofiberscopy under local anesthesia with 4% xylocaine. The tumor site was irradiated endoscopically using a 664-nm wavelength laser beam and a directional quartz fiber (straight type power density, 150 mW/cm^2^ and energy level, 100 J/cm^2^; radial type power density, 90–120 mW/cm^2^ and energy level, 100 J/cm^2^). The tip of the quartz fiber was maintained at a distance of 1–2 cm from the lesion for surface irradiation (straight-type tip) or inserted a few times into the obstructive tumor (radial-type tip; interstitial irradiation). Depending on the tumor size, both irradiation methods were used.

We performed chemotherapy (platinum-doublet or titanium silicate [TS-1]) on the day of or the day following PDT. One week and one month after PDT, the bronchi and area of the necrotic tumor were monitored, and the combination of PDT and chemotherapy was repeated. We repeated PDT for patients who were effective, had a risk for restenosis, or had a performance status of one or two.

### 3.4. Evaluation of Effectiveness

Treatment effectiveness was assessed using symptom relief, airway secretion aspirate, bronchoscopic evidence of alteration in the luminal obstruction, which is expressed as a percentage of the total bronchial luminal diameter, and evidence of removal of the tumor necrosis.

The tumor response was evaluated based on computed tomography (CT), which was conducted at baseline and again after two cycles of first-line PDT with chemotherapy. Experienced investigators recorded the measurement of target lesions, development of new lesions, and tumor response for each patient according to the Response Evaluation Criteria In Solid Tumors (RECIST) 1.1. To estimate the efficacy of PDT, pulmonary function tests were conducted with three patients before and after PDT. QOL was assessed using the KS before and one month after treatment.

### 3.5. Statistical Analyses

Values are presented as median (range). Paired *t*-tests were used for comparisons. The Kaplan-Meier method was used to create survival curves. *p* < 0.01 was considered significant, and the analyses were conducted using JMP version 10 (SAS Institute, Tokyo, Japan).

## 4. Conclusions

PDT appears to be efficacious in the treatment of patients with advanced lung cancer with airway stenosis. Following treatment with PDT, all of the patients experienced improved symptoms and QOL, in as early as one week. In addition, objective response was indicated by the substantial increase in the openings of the bronchial lumen and the prevention of obstructive pneumonia. Furthermore, the combination of PDT with standard treatment methods appears to be safe, without additional toxicity, even in patients with complications such as ILD.
